# Supplementary material for: Architecture of the RNA polymerase II-Paf1C-TFIIS transcription elongation complex
Source: Nat Commun. 2017 Jun 6;8:15741. doi: 10.1038/ncomms15741 (PMC5467213; doi:10.1038/ncomms15741)
Supplement: Supplementary Information — Supplementary Figures [file ncomms15741-s1.pdf]

SUPPLEMENTARY FIGURES

Supplementary Figure 1:

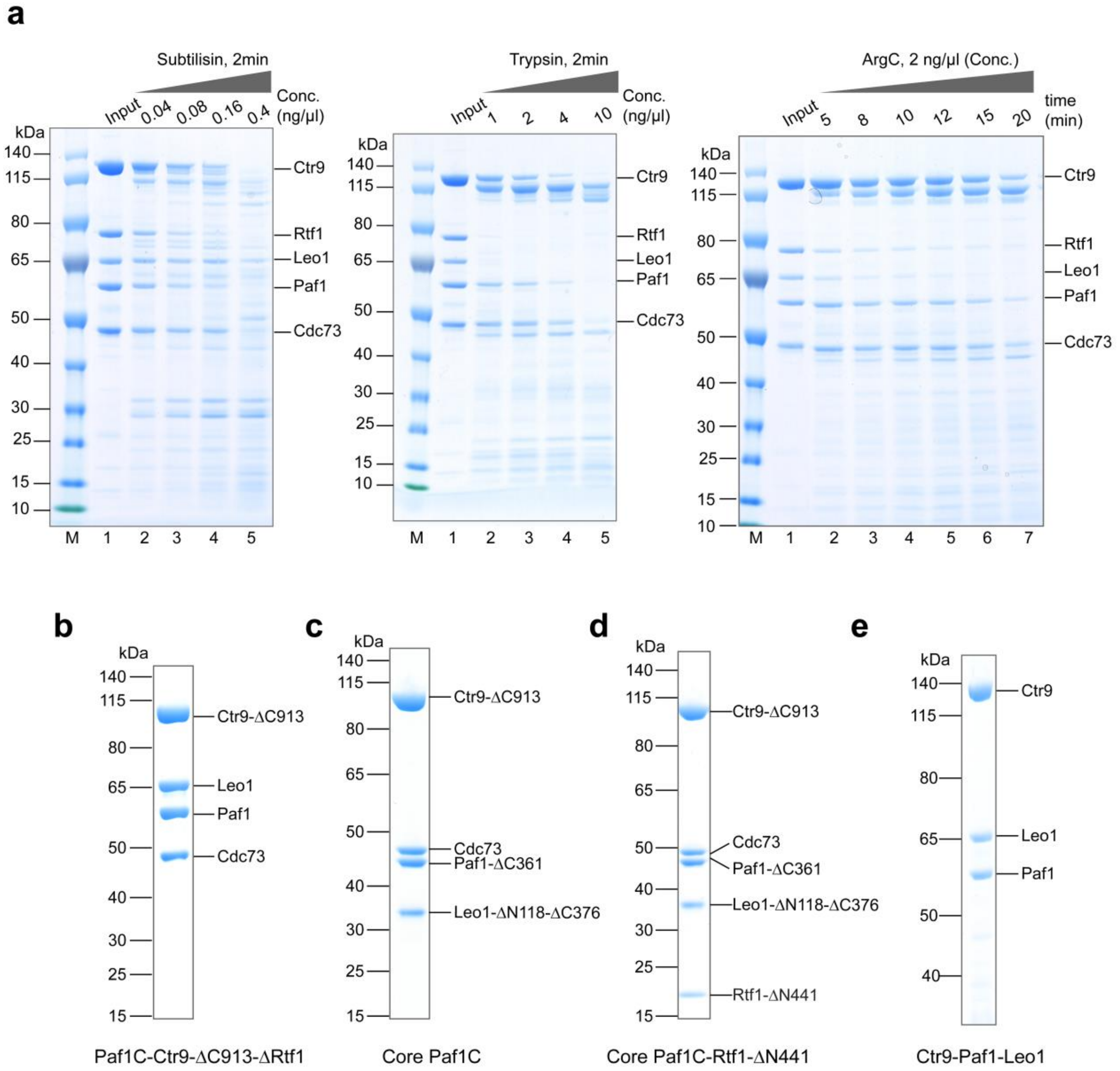

Supplementary Figure 1 Preparation of various Paf1C recombinant proteins.

a Limited proteolysis using subtilisin, trypsin, and ArgC under either various concentrations or reaction times shows Paf1C contains many flexible regions.

b-e Coomassie-stained SDS-PAGE analysis of (**b**) Paf1C-Ctr9-ΔC913-ΔRtf1, (**c**) core Paf1C, (**d**) core Paf1C-Rtf1ΔN441, and (**e**) Ctr9-Paf1-Leo1 after size-exclusion chromatography (Superose 6 10/300; GE Healthcare) reveals three-subunit, four-subunit, and five-subunit Paf1C variants. Molecular weight markers are indicated on the left. The identity of subunits was confirmed by mass spectrometry.

Supplementary Figure 2:

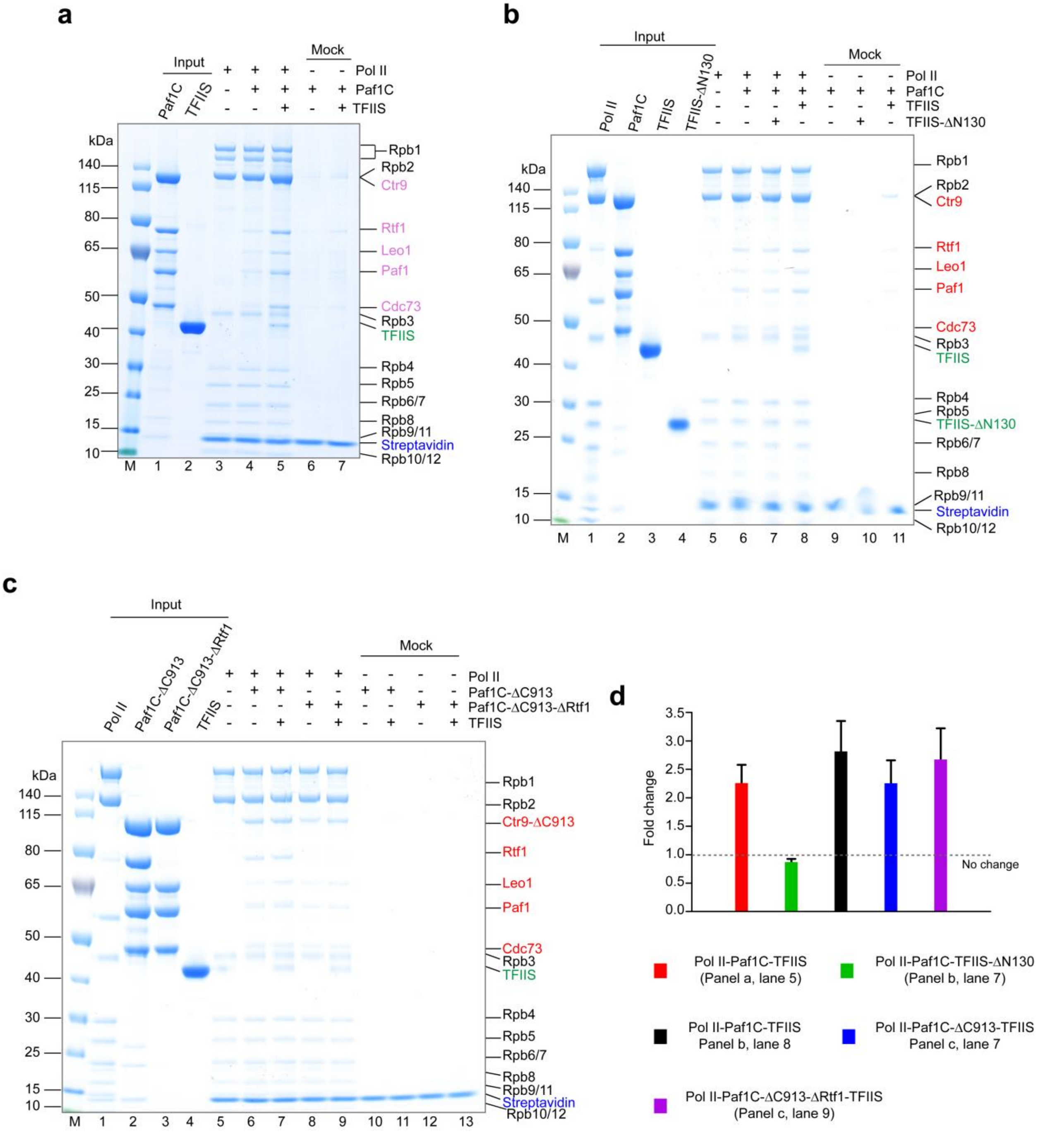

Supplementary Figure 2 Analysis of Pol II-Paf1C -TFIIS interactions.

SDS-PAGE analysis of Pol II-Paf1C-TFIIS binding assays. Subunits from Pol II, Paf1C, and TFIIS are labeled in black, pink, and green, respectively.

a TFIIS enhances Pol II-Paf1C binding. Lane 4 and 5 show Pol II-Paf1C and Pol II-Paf1C-TFIIS interactions, respectively. When TFIIS is present, Pol II and Paf1C bind better.

b TFIIS, but not TFIIS-ΔN130, cooperatively binds to Pol II-Paf1C (lane 7 and 8).

c Paf1C lacking Rtf1 and the C-terminal domain of Ctr9 retains Pol II binding (lane 6 and 8) and TFIIS-enhanced binding (lane 7 and 9).

d Relative binding fold changes between Pol II and Paf1 complexes when TFIIS or TFIIS-ΔN130 presents. Rpb4 is used to normalize lane-specific effects to make lanes comparable. Set to 1 in cases lacking TFIIS (representing unchanged amounts). Error bars indicate the standard error of the mean from 3 replicates.

Supplementary Figure 3:

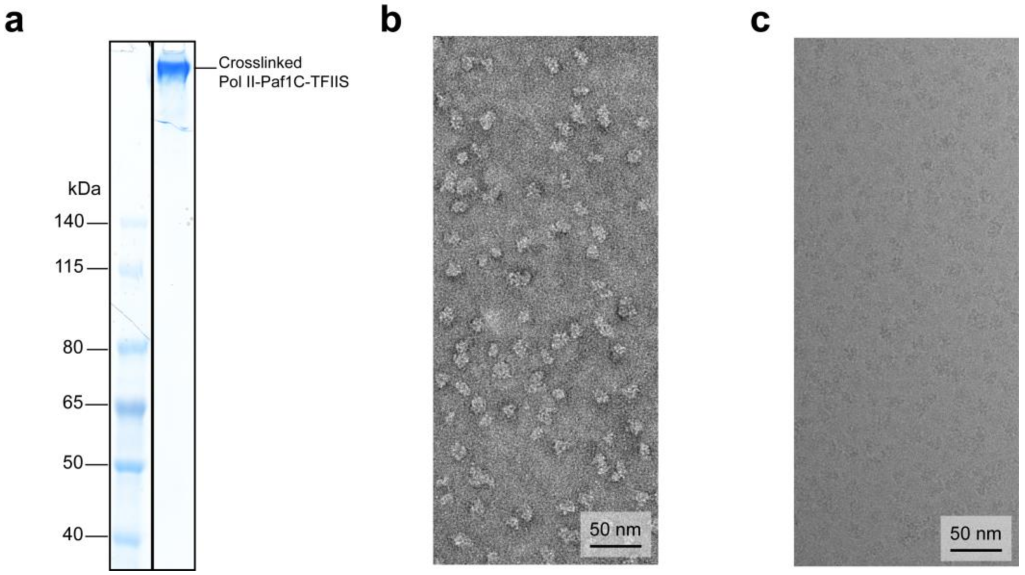

Supplementary Figure 3 Pol II-Paf1C-TFIIS EC sample preparation for cryo-EM and a representative cryo-EM micrograph.

a SDS-PAGE analysis of crosslinked Pol II-Paf1C-TFIIS EC after sucrose gradient fixation. A shift to higher molecular weight indicates successful crosslinking.

b A negative stain micrograph of Pol II-Paf1C-TFIIS with 50 nm scale bar included as a size reference showing a good quality of Pol II-Paf1C-TFIIS EC sample. This micrograph was obtained using an FEI Philips CM-200 at 160 kV and a magnification of 88,000x with a defocus ~2.0 µm.

c A representative cryo-EM micrograph of Pol II-Paf1C-TFIIS EC with 50 nm scale bar included as a size reference. This movie stack was collected on a 300 keV FEI Titan Krios electron microscope equipped with a K2 Summit direct electron counting camera (Gatan) positioned post a GIF Quantum® energy filter (Gatan) at a nominal magnification of 37,000x. An exposure time of 12 s fractionated into 30 frames in this stack and a total accumulated dose of approximate 28 electrons per Å2 per stack were used. The defocus value of this stack was -1.5 µm.

Supplementary Figure 4:

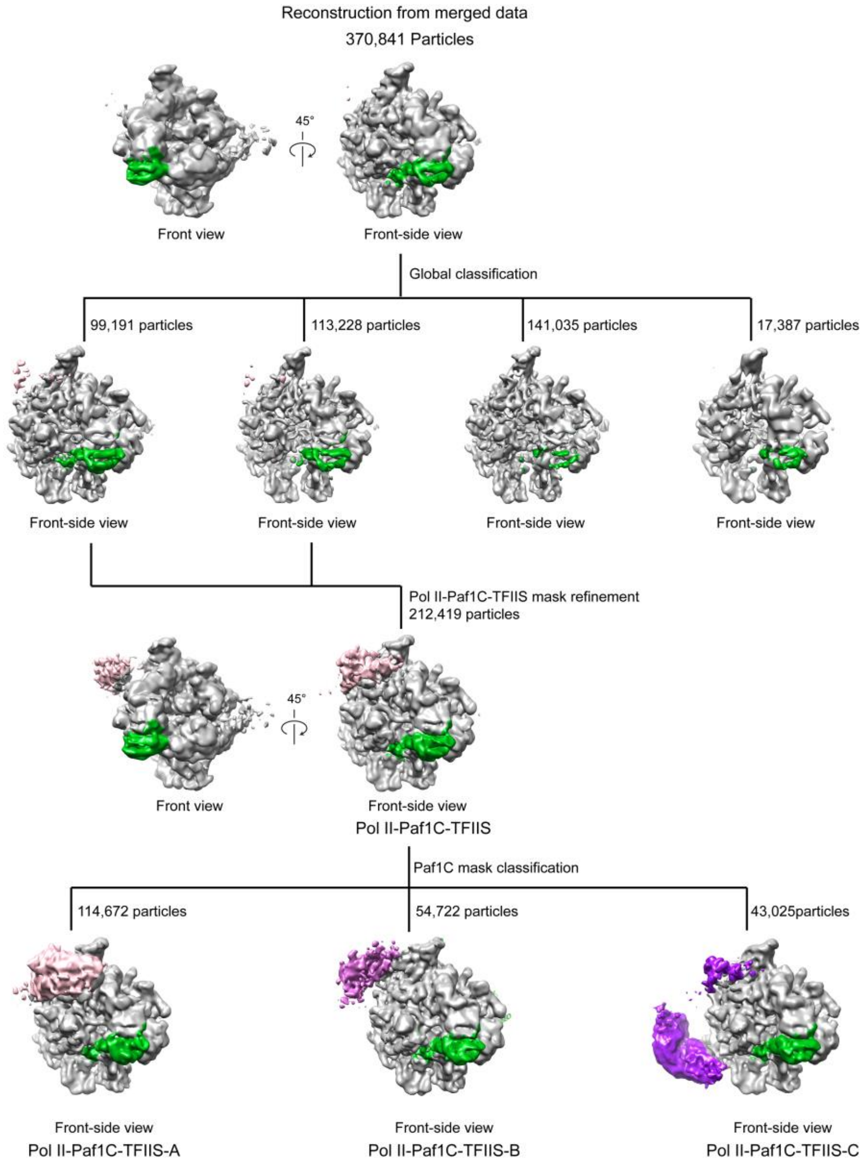

Supplementary Figure 4 Computational sorting of cryo-EM particle images.

Particles were 3D-classified in RELION to reveal additional features of Pol II-Paf1C-TFIIS after 2D classification. Classifications were performed without image alignment by global 3D classification using a spherical mask. After global classification, we separated Pol II-Paf1C-TFIIS and Pol II-TFIIS complexes. We further merged two classes with additional density near the protrusion. After auto-refinement and subsequent 3D classification with a mask encompassing Paf1C, we obtained three structures displaying different parts of Paf1C. Pol II and scaffold are colored in grey, whereas TFIIS is in green. Parts A, B, and C of Paf1C are in pink, orchid and purple, respectively. To visualize the density from Paf1C, a front-side view as in Fig. 3 is used.

Supplementary Figure 5:

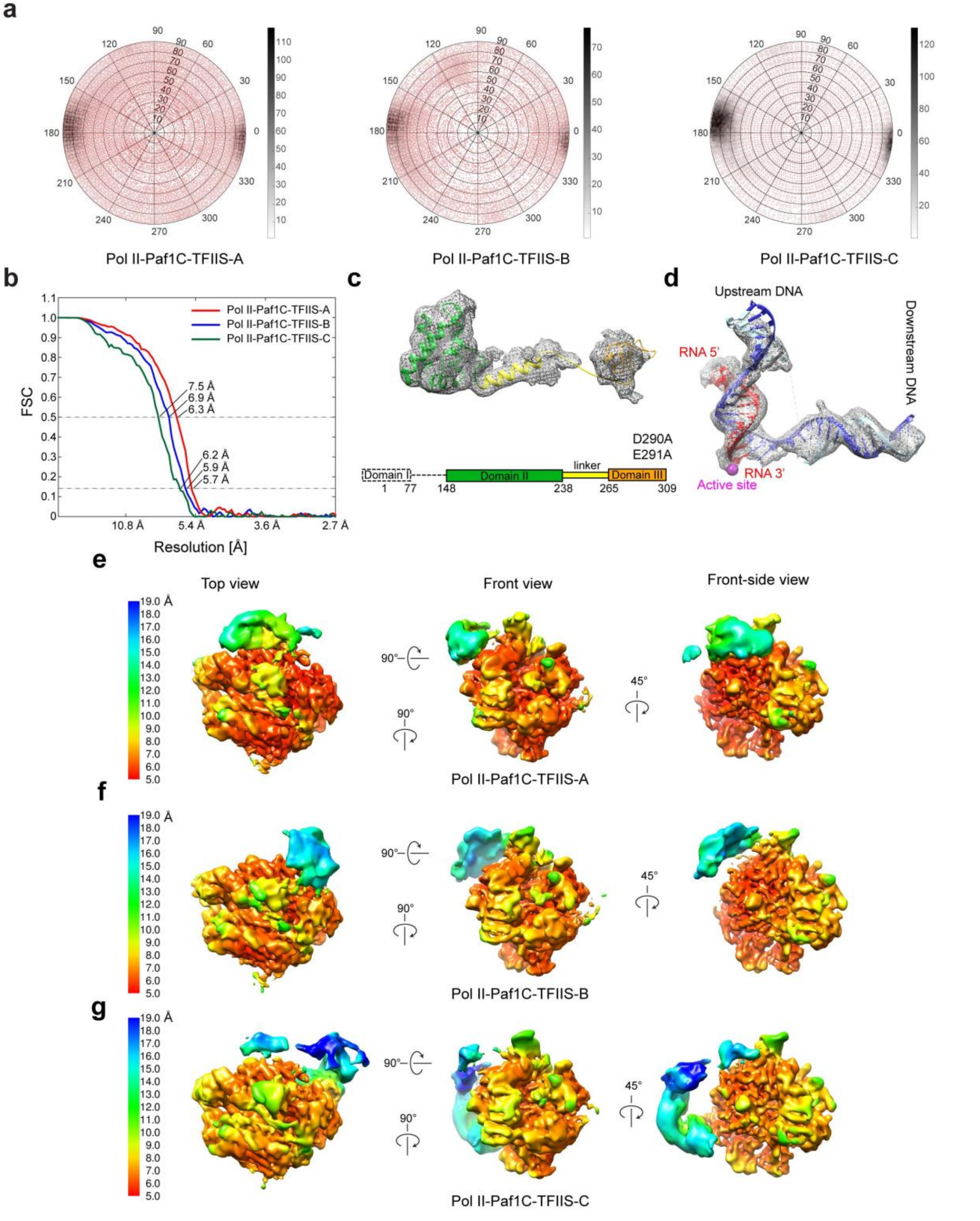

Supplementary Figure 5 Cryo-EM reconstruction of Pol II-Paf1C-TFIIS subclasses.

a Angular distribution of Pol II-Paf1C-TFIIS reconstructions A, B, and C from cryo-EM single particle reconstructions. Red dots indicate at least one particle was assigned within 1° of the point. Black shading indicates the number of particles assigned to a given view.

b The FSC curves for the Pol II-Paf1C-TFIIS subclasses. Color scheme: Pol II-Paf1C-TFIIS reconstruction A, red; reconstruction B, blue; reconstruction C, dark green. The resolution of the reconstructions using the Fourier shell cutoff at 0.5 and 0.143 is shown.

c TFIIS EM density (grey mesh) from the unsharpened Pol II-Paf1C-TFIIS. Crystal structure derived from 3PO3 is fitted. A schematic diagram of TFIIS domains is shown below. Domain II, linker, and domain III are represented in green, yellow, and orange boxes. Point mutations of two functionally essential acidic residues are indicated. No density was observed for TFIIS residues 1-148 containing domain I and a linker, which are labeled with dashed lines due to their flexibility.

d EM density for the DNA-RNA hybrid and up- and downstream DNA of Pol II-Paf1C-TFIIS (grey mesh) with the final model superimposed. Color scheme: non-template DNA, cyan; template DNA, blue; RNA, red; active site, magenta.

e-g Top, front and front-side views of unsharpened (**e**) Pol II-Paf1C-TFIIS reconstruction A, (**f**) reconstruction B, (**g**) reconstruction C colored by local resolution. All the densities are shown at the same threshold level and locally normalized. Resolution bars are shown on the left.

Supplementary Figure 6:

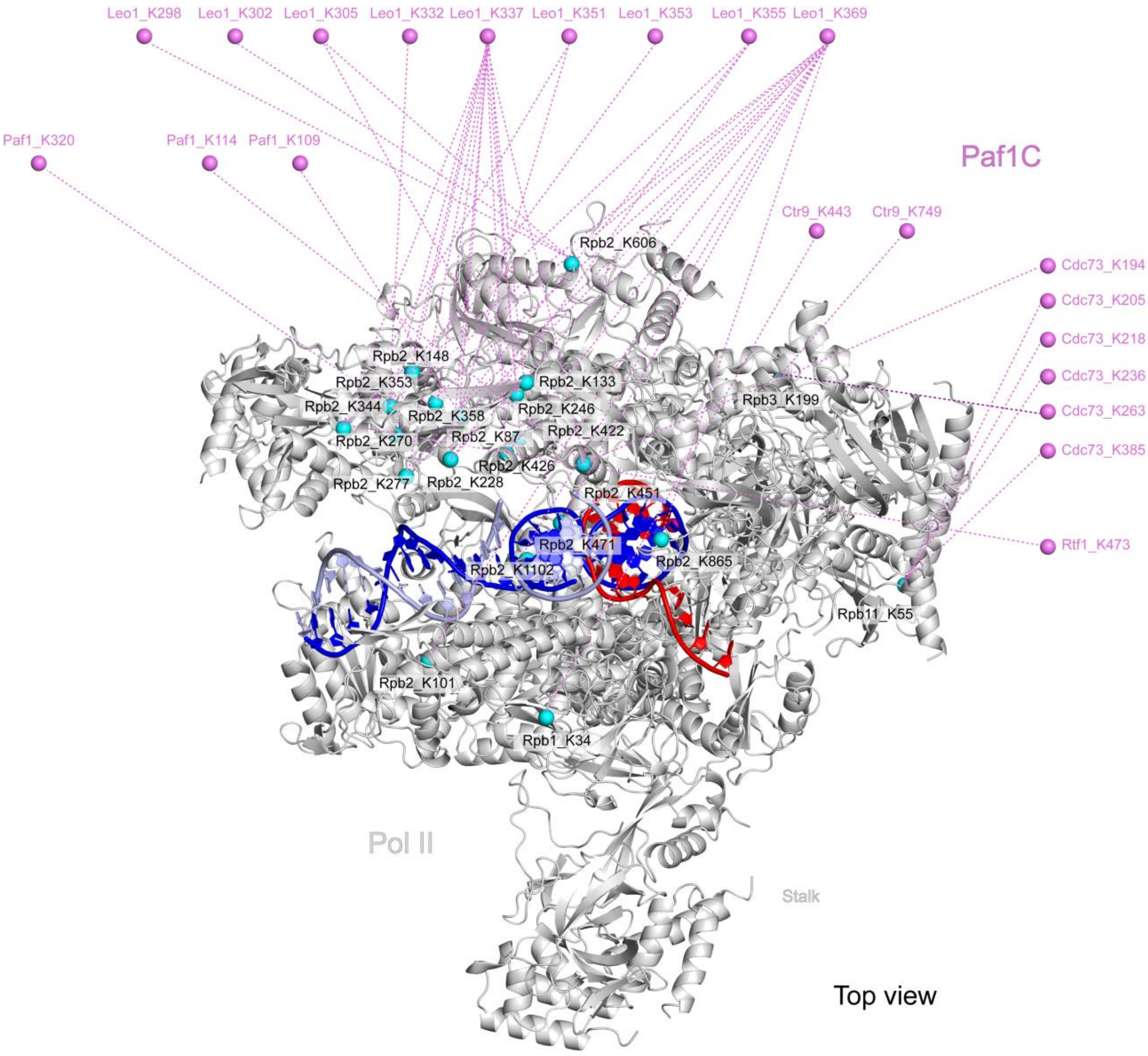

Supplementary Figure 6 Regions on the Pol II-TFIIS EC surface that crosslink to Paf1C. The crosslinked lysine residues on Pol II-TFIIS EC structure are highlighted in cyan spheres. The crosslinked lysine residues from Paf1C are represented with pink spheres. The dash lines represent the crosslinks between lysine-lysine pairs. Pol II-TFIIS EC structure is colored in grey and shown in a top view. Color scheme: non-template DNA, light blue; template DNA, blue; RNA, red.
